# Comparison of Phenolic Acids and Flavan-3-ols During Wine Fermentation of Grapes with Different Harvest Times

**DOI:** 10.3390/molecules14020827

**Published:** 2009-02-18

**Authors:** Rong-Rong Tian, Qiu-Hong Pan, Ji-Cheng Zhan, Jing-Ming Li, Si-Bao Wan, Qing-Hua Zhang, Wei-Dong Huang

**Affiliations:** College of Food Science and Nutritional Engineering, China Agricultural University, Beijing, 100083, P.R. China; E-mails: rongx2tian@yahoo.com.cn (R-R.T.), panqh@126.com (Q-H.P.), zhangjich@126.com (J-C.Z.), lijingm@126.com (J-M.L.), wansib@126.com (S-B.W.), zhangqihua@126.com (Q-H.Z.)

**Keywords:** Phenolic acids, Flavan-3-ols, Harvest times, Wine.

## Abstract

To explore the effects of harvest time on phenolic compounds during wine fermentation, grape berries (*Vitis vinifera* L. cv. Vidal) were harvested at 17.5, 22.8 and 37.2º Brix and were used to make dry wine, semi-sweet wine and icewine with low alcohol levels, respectively. Phenolic acids and flavan-3-ols were assayed during the fermentation of wines by means of reverse phase-high performance liquid chromatography (RP-HPLC). The results showed that concentrations of most of the phenolic acids and flavan-3-ol in musts increased with harvest time delay and higher total levels of these species were detected in all wines, compared with those measured before fermentation (the total phenolic acid content in wines was 1.5-2.0 fold that of in musts). Except for *p-*coumaric acid and (-)-epicatechin, other phenolic acids and flavan-3-ols had similar variation patterns (wave-like rise) during fermentation in dry wine and semi-sweet wine. However, some detected compounds, including gentisic acid, *p-*hydroxybenzoic acid, caffeic acid, *p-*coumaric acid and sinapic acid showed obviously different trends from the other two wines in the icewine making process. It is thus suggested that the harvest time has a decisive effect on phenols in final wines and influences the evolution of phenolic acids and flavan-3-ols during wine fermentation.

## Introduction

Phenolic compounds are abundant in wines and play an important role in controlling oxidation in the human body. They possess reported to have anticancer and anti-inflammatory effects *in vitro*, as well as the ability to block cellular events predisposing to atherosclerosis and coronary heart disease (CHD) [1,2,3,4,5]. A large number of published works have focused on the essential contributions of phenolic profiles to wine quality and sensory properties [6,7,8]. Generally, phenolic compounds in wine are divided into colored phenols, such as anthocyanins and flavonoids, and non-colored phenols, including phenolic acids and flavan-3-ols. Non-colored phenols are a group of important secondary metabolites in white grape berries and responsible for the bitter and astringent properties of wine [7,8,9,10]. The phenolic profiles in wine depend on the phenols contained in the grapes, the extraction parameters, wine making technologies as well as chemical reactions taking place during wine fermentation and ageing [11,12,13,14,15,16], while the phenolic compounds of grapes are affected by many factors, including variety, maturity, climatic and geographical conditions [17,18,19,20]. The effect of maturity degree and harvest times on phenolic levels in grapes and wines has attracted great attention [11,14,19,21]. In general, the content of phenolic compounds increases throughout ripening of grapes and the concept of “phenolic maturity” has even been introduced in recent years [21]. Ramos had also revealed that 3, 4-dihydroxybenzoic acid content apparently increased with a 15-day harvest time delay [14]. There are different evolutions in different phenolic families corresponding to the increased degree of maturity [21,22,23], but previous reports have focused intensely on changes of phenolic compounds in ripe berry-fermented wine. Little is reported concerning composition of phenols during winemaking using over-ripened (or late harvest) berries as materials. Moreover, in contrast to some reports about phenolics during red wine fermentation, especially malolactic fermentation [14,15], little attention seems to have been paid to the evolution of phenolic compounds during alcoholic fermentation of white wine. 

Vidal is a typical white grape cultivar, which is often used for the production of dessert wines such as icewine due to the fact that the berries have relatively thick skins and the vines are cold resistant [24,25]. In the present study, Vidal grape berries were harvested at full ripeness and at one month after that, respectively, to make dry wine and semi-sweet wine with low alcohol degree and different residual sugar. The icewine was made when the frozen grapes reached the production parameters for authentic icewine set out by the Vintners Quality Alliance (VQA) in Canada. According to these, the grape berries for making icewine must be harvested at lower than -8 °C, and pressed in the frozen state to release the very concentrated juice, which is required to be a minimum of 35º Brix prior to fermentation [26]. Here, phenolic profiles in various wines from berries of different harvest times were investigated, the objective of which is to explore the influence of harvest times to the phenols during the process of fermentation.

## Results and Discussion

**Figure 1:**
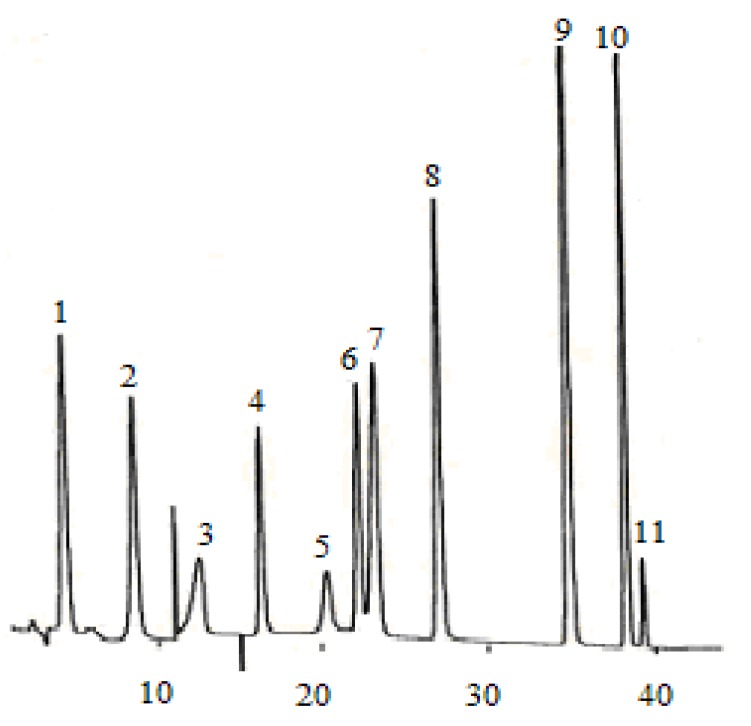
Chromatogram of 11 phenolic acid standards. (1) gallic acid, (2) protocatechuic acid, (3) gentisic acid, (4) *p-*hydroxybenzonic acid, (5) chlorogenic acid, (6) vanillic acid, (7) caffeic acid, (8) syringic acid, (9) *p-*coumaric acid, (10) ferulic acid, (11) sinapic acid.

According to the chromatographic separation method described, the chromatograms obtained for eleven phenolic acids and five flavan-3-ols are shown in Figure 1 and Figure 3, while the phenolic acids and flavan-3-ols detected in wine samples are shown inFigure 2 and Figure 4.

**Figure 2:**
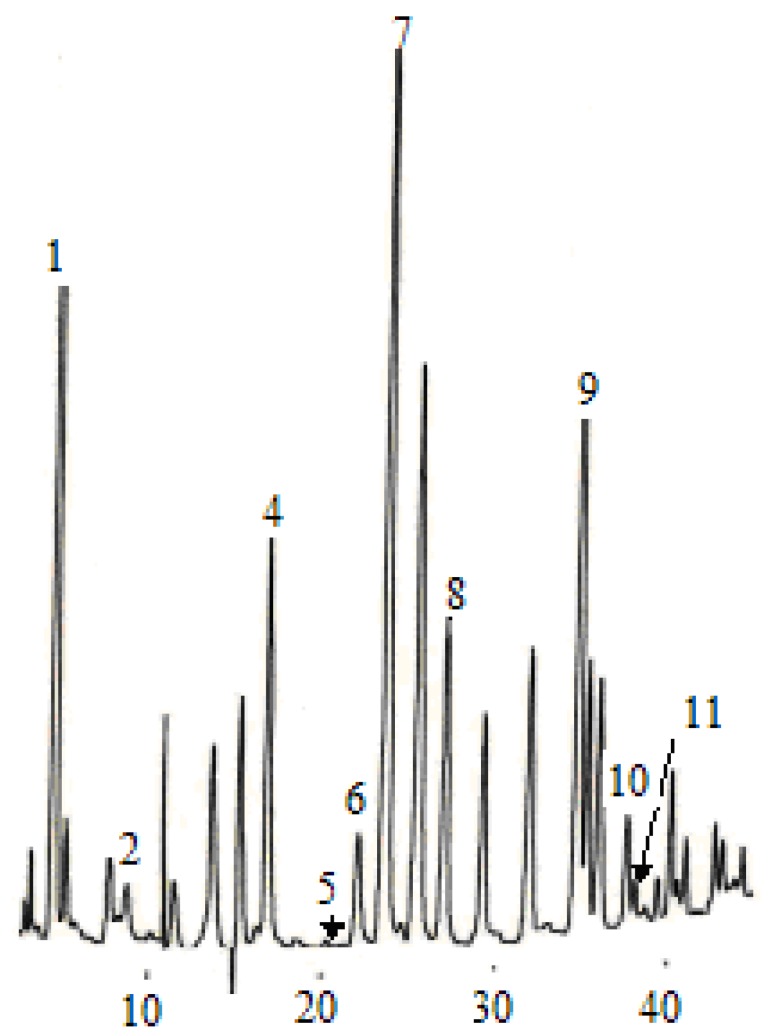
Phenolic acid compounds detected in the dry wine sample. (1) gallic acid, (2) protocatechuic acid, (4) *p-*hydroxybenzonic acid, (5) chlorogenic acid, (6) vanillic acid, (7) caffeic acid, (8) syringic acid, (9) *p-*coumaric acid, (10) ferulic acid, (11) sinapic acid.

**Figure 3:**
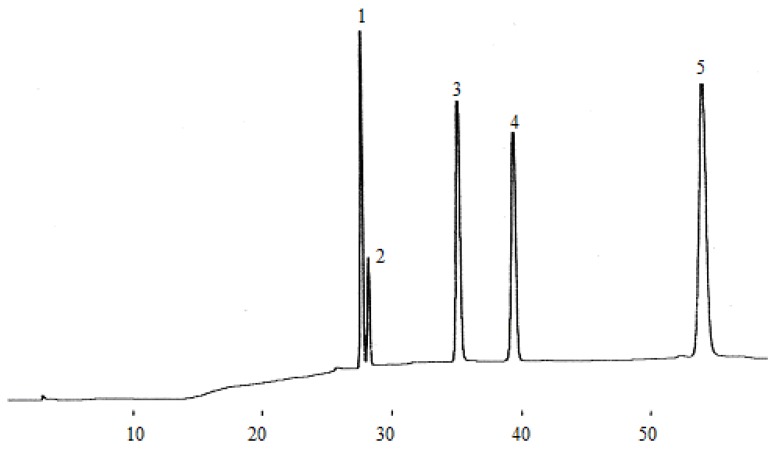
Chromatogram of five flavan-3-ol standards. (1) catechin, (2) epigallocatechin, (3) epigallocatechin galloate, (4) epicatechin, (5) epicatechin galloate.

**Figure 4:**
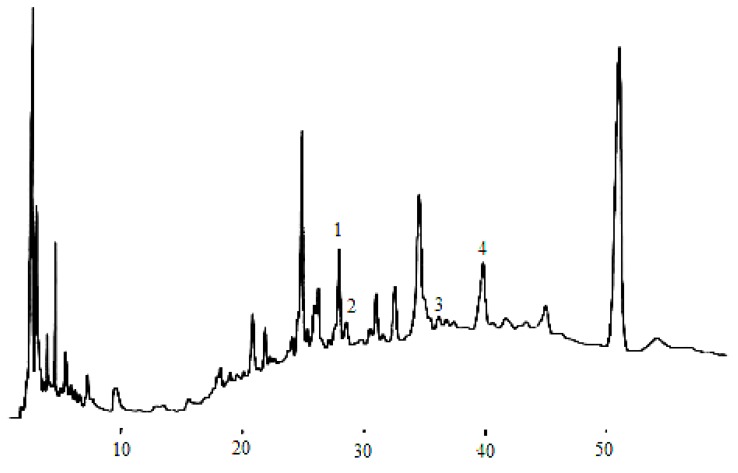
Flavan-3-ol compounds detected in the dry wine samples. (1) catechin, (2) epigallocatechin, (3) epigallocatechin galloate, (4) epicatechin.

### Comparison of phenolic acids during the fermentation of various wines

The three kinds of musts with significant differences in sugar levels were fermented at controlled temperature (13 ± 2 °C), respectively, and differences in the residual sugar of the resulting wines with similar alcohol degrees (9.8-10.4%) are shown in Table 1. These wines were called dry wine, semi-sweet wine and icewine (also, sweet wine), respectively.

**Table 1:** Mean values of parameters for wines before and after fermentation.

	Dry wine		Semi-sweet wine		Icewine (sweet wine)	
	Before fermentation	After fermentation	Before fermentation	After fermentation	Before fermentation	After fermentation
Sugar (mg/L)	175.41	2.20	228.33	44.25	372.45	197.24
Ethanol (%, v/v)	undetected	9.8	undetected	10.4	undetected	10.0

**Figure 5:**
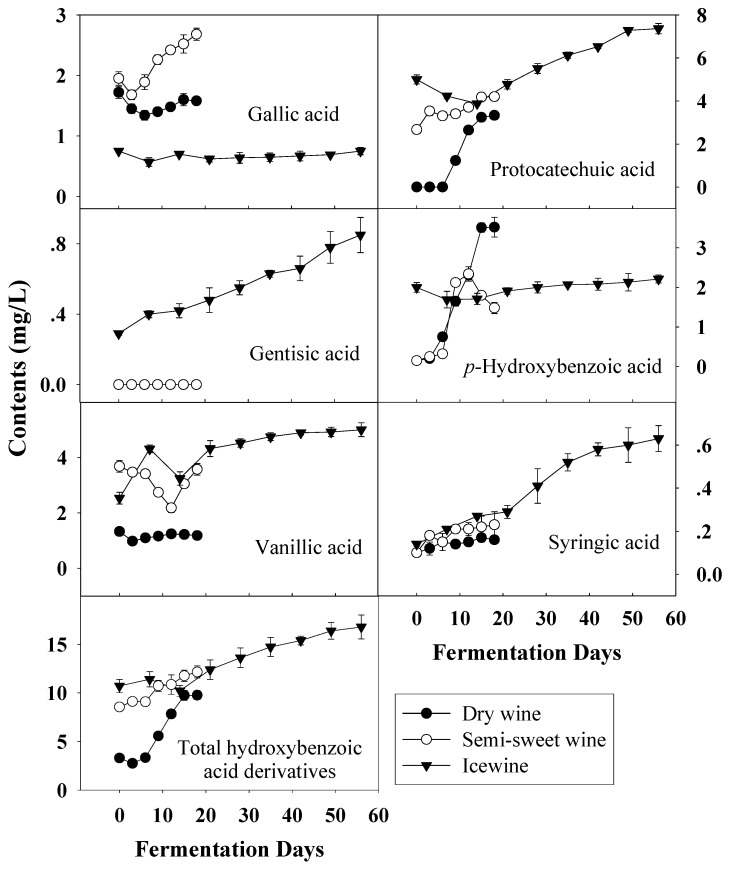
Evolution of hydroxybenzoic acid derivatives durin the fermentation of dry wine, semi-sweet wine and icewine.

In wine, there are two groups of phenolic acids; hydroxybenzoic acids and hydroxycinnamic acids [27]. Hydroxybenzoic acids, including gallic acid, protocatechuic acid, gentisic acid, *p-*hydroxybenzoic acid, vanillic acid and syringic acid, derive from benzoic acid. Figure 5 shows the evolution of hydrobenzoic acids during the fermentation of dry wine, semi-sweet wine and icewine. In terms of total hydroxybenzoic acids, the total concentration in the musts increased as harvest times were delayed and higher levels were observed in all wines, compared with those before fermentation. 

Of the six hydroxybenzoic acid derivatives measured, only the gallic acid content was lower in frozen grape musts than in the other two, and it remained at a low level during the whole fermentation process. This may be because more gallic acid existed in the form of tartaric acid esters with the extended ripening. Syringic acid content showed little difference among the three kinds of musts and similar syringic acid changes were also observed throughout the fermentation of all wines. The concentration of protocatechuic acid, which was predominant in all wines, increased continuously during fermentation and showed the same trend as that of *p-*hydroxybenzoic acid and syringic acid.

**Figure 6:**
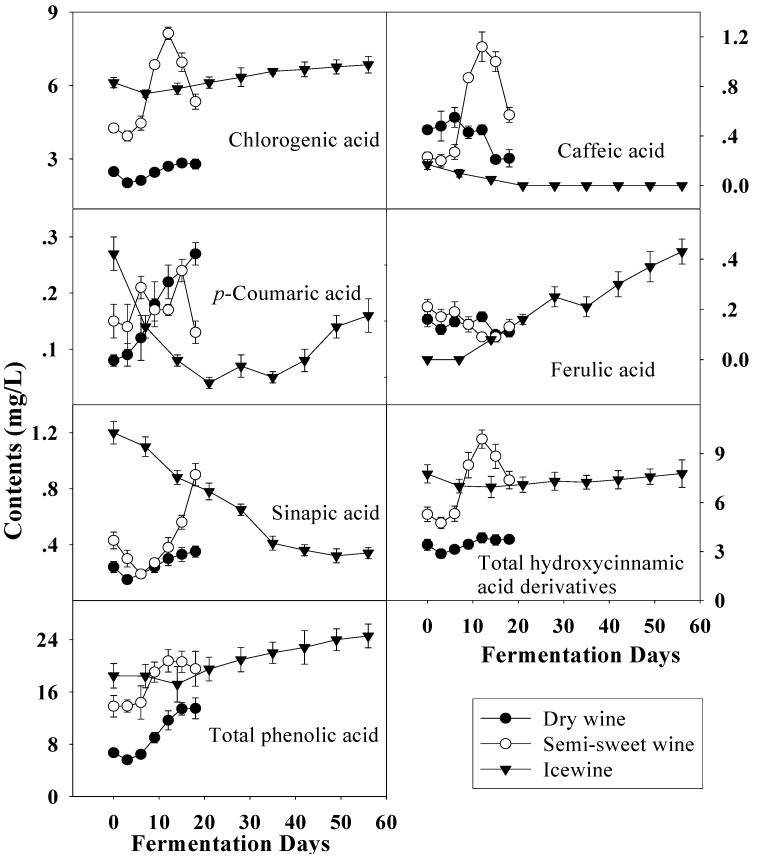
Evolution of hydroxycinnamic acid derivatives and total phenolic acid during the fermentation of dry wine, semi-sweet wine and icewine.

With harvest time delay the total level of hydroxycinnamic acid derivatives, another group of phenolic acids, increased in the musts, similar to the total hydroxybenzoic acid derivatives (Figure 6). Compared to the hydroxybenzoic acid derivatives, the contents of hydroxycinnamic acid derivatives varied more gradually and were a little higher when fermentation finished. Therefore, the variation of total phenolic acid was similar to that of total hydroxybenzoic acid derivatives and rise with the fermentation. Chlorogenic acid, as the main constituent of the hydroxycinnamic acid derivative group, increased with harvest time delay, and the same occurred with sinapic acid, while the converse was the true of caffeic acid and ferulic acid, which were also esterified with tartaric acid as the known compounds caftaric acid and fertaric acid, respectively [15,27]. Compared to before fermentation, the contents of most hydroxycinnamic acids, except sinapic acid, did not show much differences in these wines and similar evolutions of the hydroxycinnamic acids during fermentation were noted in dry wine and semi-sweet wine. 

### Comparison of flavan-3-ols during the fermentation of various wines

**Figure 7:**
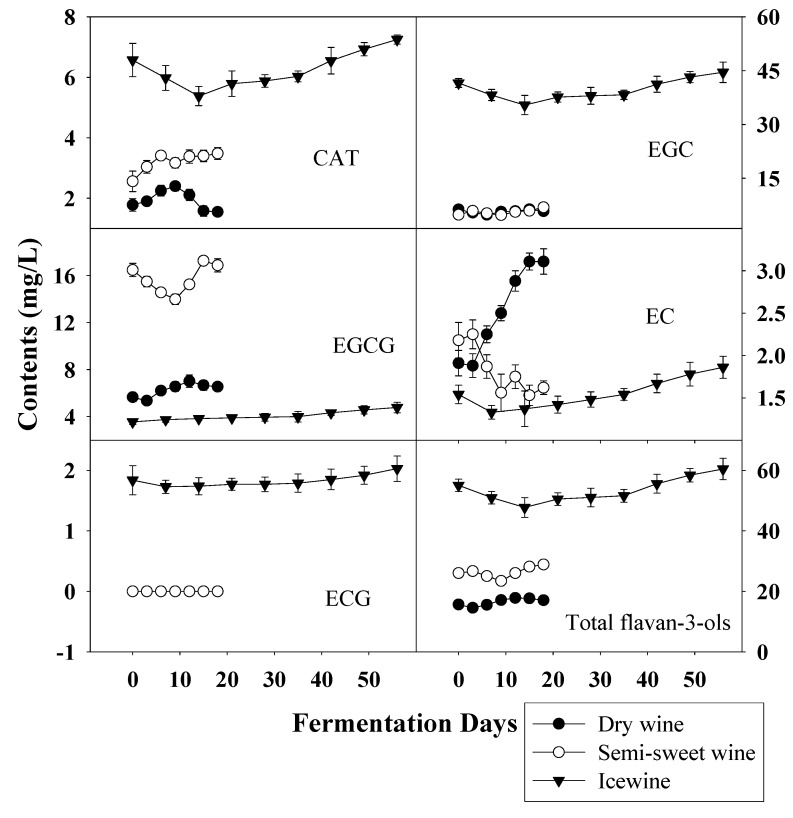
Evolution of flavan-3-ols during the fermentation of dry wine, semi-sweet wine and icewine.

In this work, flavan-3-ols, including (+)-catechin (CAT), (-)-epicatechin (EC), (-)-epicatechin gallate (ECG), (-)-epigallocatechin (EGC), (-)-epigallocatechin gallate (EGCG) were all detected by RP-HPLC. As shown in Figure 7, total flavan-3-ol in frozen grape musts was much higher than that in other musts and no obvious trend was observed during the fermentation. However, the evolutions of individual flavan-3-ols were different obviously from that of phenolic acids and the contents remained steady during the whole fermentation.

The contents of most flavan-3-ols, except EC and EGCG, were all much higher during the whole fermentation of icewine than the others, especially the concentration of EGC in icewine was 7.6-fold that in dry wine and 6.5-fold that in semi-sweet wine. However, the content of EGCG in icewine was at a very low level (4.78 ± 0.44 mg/L), only accounting for 0.08% of the total content, while EGCG in semi-sweet wine was the main constituent of the flavan-3-ols, being 58.45% of the total. For dry wine, only the content of EC was much higher than the other wines, but the level was low (3.11 ± 0.15 mg/L). These variations were assumed to be due to the different transformations of phenolic compounds, such as condensation and polymerisation reactions involving individual flavan-3-ols, and these reactions are related to sensorial characteristics of the final wines [3]. In fact, flavan-3-ols are found in the skin and seed tissue and abundant in red wine [15,18], while a low relatively level is found in white wine [28].

It is documented that the phenolic content generally increases throughout the ripening of grapes [21], which was coincident with our research. During the vinification of grapes, wine-making process, together with the effect of other parameters such as temperature, species of yeast strains and stirring of the fermentative medium has a considerable influence on the phenolic variation during fermentation and final phenolic content of wine [6,29,30,31]. In our study, the same temperature and commercial yeast, eliminating the effects of temperature and yeast, were used in the fermentation of all wines and the whole of fermentation was strictly controlled at the same conditions. Moreover, the incomplete fermentation in semi-sweet wine and icewine kept the alcohol at the level 9-11% (v/v) in all wines. Thus the influences of harvest time on wine fermentation were maximized to explore better the effects of maturity degree on the phenols during the process of fermentation. Overall, as shown in Figure 5, Figure 6 and Figure 7, harvest times (directly correlated with degree of maturity of grapes) strongly influenced the evolution of phenolic acids and flavan-3-ols during the fermentation of wines and even had a decisive effect on final phenolic profiles in wines. 

## Experimental

### Plant material

All the berries (*Vitis vinifera* L. cv. Vidal) were supplied by the commercial vineyard (about 4.0 ×10^6^ m^2^) in Beidianzi Village, Huanren County, Liaoning Province, P. R. China. The grape planting and management process strictly obeyed the planting techniques of Liaoning Changyu Icewine Chateau Co., Ltd. Each grape sample was randomly selected from different grapevines located in different rows on the basis of similar size and absence of physical injuries or infections.

### Wine samples

The grapes were harvested on Oct. 25, 2005, Nov. 26, 2005 and Feb. 12, 2006. After crushing, each must sample, 300 liters were divided into three equal parts in tanks with a volume of 100 L to make the dry wine, semi-sweet wine and icewine, respectively. Due to the accumulation of sugars during grape ripening, the total sugars given by the grapes used in this work are shown in Table 1. The fermentations were controlled at 13 ± 2 °C using the same commercial yeast (R2, purchased from Lallemand Inc., Blagnac, France; 30 g/hL musts), respectively. The fermentations of the semi-sweet wine and icewine were both incomplete, leaving some residual sugar and were stopped by low temperature and adding SO_2_. In the juice used for making icewine, the sugar content was so high that the fermentation was very slow. Up to 60 days, the alcohol level was similar to that in dry and semi-sweet wine. In all cases, the samples were collected at 3 (for dry wine and semi-sweet wine) or 7 (for icewine) day intervals during fermentation for analysis samples. Three representative samples were taken from each kind of wine during fermentation and phenolic acids and flavan-3-ols from each sample were analyzed in triplicate. Results presented in the figures are shown with standard deviations and the arithmetic mean of the nine analyses.

### Standards

Phenolic acid standards, including gallic acid, protocatechuic acid, *p-*hydroxybenzonic acid, syringic acid, chlorogenic acid, caffeic acid, *p-*coumaric acid and sinapic acid were purchased from Sigma Chemical Co. (St. Louis, USA), and gentisic acid, vanillic acid and ferulic acid were purchased from Fluka (Buchs, Switzerland). Flavan-3-ols standards, including (+)-catechin (CAT), (-)-epicatechin (EC), (-)-epigallocatechin (EGC), (-)-epicatechin gallate (ECG) and (-)-epigallocatechin gallate (EGCG) were purchased from Sigma Chemical Co. (St. Louis, USA). The purities of the 16 standards were all over 95%. HPLC grade methanol was purchased from Fisher Scientific (Pittsburgh, PA, USA) and used as a solvent.

### Determination of phenolic acid and flavan-3-ol content

Without any extraction or hydrolysis, all wine samples were filtered through a 0.45 µm filter (Acrodisc LC13 PVDF filter, Gelman/Pall Life Sciences, MI) prior to injection onto the HPLC for analysis (10 µL per injection). The compounds detected were identified by the comparison with their retention times with those of pure standards. The HPLC system consisted of a Shimadzu (Japan) LC-6A series pumping system, SIL-6A automatic injector furnished with a 50-µL loop, SPD-6AV UV–visible detector and CR6A chromatography data station software. Chromatographic separations were performed on a Merck LiChrospher 100RP-18e (Merck, Germany) column (250 × 4.0 mm ID, 5 um), protected by a Merck RP-18 (10 mm × 4.0 mm) guard column. Both columns were placed in a column oven set at 30 °C. 

Gentisic acid had an elution time of between 11-15 min and was detected at 320 nm, and the other 10 phenolic acids were detected at 280 nm. Solvent A consisted of 10% methanol and 2% acetic acid in water, and solvent B included 90% methanol and 2% acetic acid in water. For the elution program, the following proportions of solvent B were used: 0-25 min, 0-15% B; 25-45 min, 15-50% B; 45-53min, 50-0% B at a flow rate of 1.0 mL/min [32]. 

Flavan-3-ols were detected at 280 nm. Two solvents were used with a constant flow rate of 1.0 mL/min. Solvent A was water and solvent B was 90% acetic acid in water. For the elution program, the following proportions of solvent B were used: 0-20 min, 7.5-65% B; 20-30 min, 65-80% B; 30-48min, 80-90% B; 48-55min, 90% B; 55-63min, 90-7.5% B. 
